# Association of electronic cigarette exposure with serum uric acid level and hyperuricemia: 2016-2017 Korea National Health and Nutritional Examination Survey

**DOI:** 10.1371/journal.pone.0247868

**Published:** 2021-03-01

**Authors:** Taeyun Kim, Yunkyung Kim, Jihun Kang

**Affiliations:** 1 Division of Pulmonology, Department of Internal Medicine, The Armed Forces Goyang Hospital, Goyang-si, South Korea; 2 Division of Rheumatology, Department of Internal Medicine, Kosin University College of Medicine, Kosin University Gospel Hospital, Busan, South Korea; 3 Department of Family Medicine, Kosin University College of Medicine, Kosin University Gospel Hospital, Busan, South Korea; Universidad Miguel Hernandez de Elche, SPAIN

## Abstract

**Objectives:**

The present study evaluated the association of electronic cigarette (EC) exposure with serum uric acid (UA) level and hyperuricemia (HUA) using a nationally representative sample of South Korea.

**Methods:**

This study included 10,692 participants (9,905, 609, and 178, never, ever, and current EC users, respectively). Urinary cotinine and 4-(methylnitrosamino)-1-(3-pyridyl)-1-butanol (NNAL) levels were used to determine conventional smoking exposure among EC users. The association between EC use and UA level was evaluated by linear regression analysis. Multivariable logistic regression analysis was used to assess the association between EC and HUA. Subgroup analysis confined to cotinine-verified active smokers was performed to address the association between the dual use of EC and combustible cigarettes and serum UA levels.

**Results:**

The serum UA level was highest among current EC users, followed by ever and never EC users. The prevalence of HUA was 26.2%, 19.3%, and 10.8% in current, ever, and never EC users, respectively. Although EC exposure was positively associated with HUA in a dose-dependent manner only in men (*P*_trend_ = 0.04), a similar tendency was also observed in women with marginal significance (*P*_trend_ = 0.102). The positive association of HUA with EC exposure was more apparent among dual users (odds ratio [OR] = 1.96, 95% confidence interval [CI]: 1.29–2.99) than among those who only smoked combustible cigarettes.

**Conclusions:**

EC exposure was associated with higher serum UA level and higher OR of HUA. The positive association between EC exposure and HUA was more prominent in dual users who concurrently consumed EC and combustible cigarettes.

## Introduction

Electronic cigarette (EC) use and the consumption of vaping products are steadily increasing, especially in never and former smokers [1]. Although ECs contain chemical components including nicotine, humectants (glycerol and glycol), heavy metals, and volatile organic compounds, they are considered less toxic than conventional cigarettes (CCs) because CCs deliver significantly higher levels of harmful and potentially harmful constituents than ECs [2]. As a result, ECs are widely used as a substitute for CCs and about 25% of current smokers use ECs as a alternative or together with CC [3]. However, since the U.S. Food and Drug Administration (FDA) reported serious adverse events of EC [4], mounting evidence has indicated that EC is associated with multiple health problems. For example, one study in healthy participants revealed that short-term exposure to EC increased respiratory flow resistance and airway resistance [5], and another study reported persistent airflow limitation and respiratory bronchiolitis after exposure to long-term use of high-dose EC [6]. Histopathological examinations showed hepatotoxicity and nephrotoxicity of ECs [7, 8].

Uric acid (UA), a metabolic derivate of purine nucleotides, is not only a main determinant for gout but is also related to several chronic diseases such as atherosclerosis, metabolic syndrome, chronic kidney disease, and hypertension [9–11]. Several studies have assessed the relationship between hyperuricemia (HUA) and CC but the results were inconsistent [12–17]. A cross-sectional study from South Korea reported that CC use was closely associated with the increased odds ratio (OR) of HUA in women; in contrast, a study by Mouhamed et al. showed a significantly lower UA level in CC users than in non-CC users [12, 13].

Although there is no clear consensus on the association between UA and gout and other chronic diseases, given the accumulating results indicating the correlation between EC use and acute and chronic disease [4, 18–20], it is important to elucidate its effect on the human body. However, compared to the volume of research on the effects of CCs on UA levels, few studies have evaluated the association between ECs and UA. Although a small study conducted in Romania showed the association between EC use and increased UA level [21], there remains a knowledge gap regarding the association between EC exposure and HUA at the population level. Nevertheless, objective measurement of the impact of ECs on HUA is challenging because many cigarette smokers use EC and CC concurrently [22]. Thus, objective methods to identify current and passive smokers such as urinary cotinine and 4-(methylnitrosamino)-1-(3-pyridyl)-1-butanol (NNAL) levels are necessary to control for potential bias due to concurrent use of CCs in studies investigating the independent health effects of ECs [23]. In this context, the present study aimed to evaluate the association between ECs with serum UA levels and HUA using population based nationally representative data.

## Materials and methods

### Study participants

This is a cross sectional study, that used data from the 2016–2017 Korea National Health and Nutritional Examination Survey (KNHANES). The KNHAHES is a nationally representative sample of non-institutionalized South Korean citizens conducted annually by the Korean Center for Disease Control and Prevention (KCDC). The KNHANES is designed as a complex sample survey by multistage stratified clustered probability sampling to represent South Korean populations. Raw data are freely accessible on the KCDC website; detailed study profiles were described previously [24].

**Fig 1:**
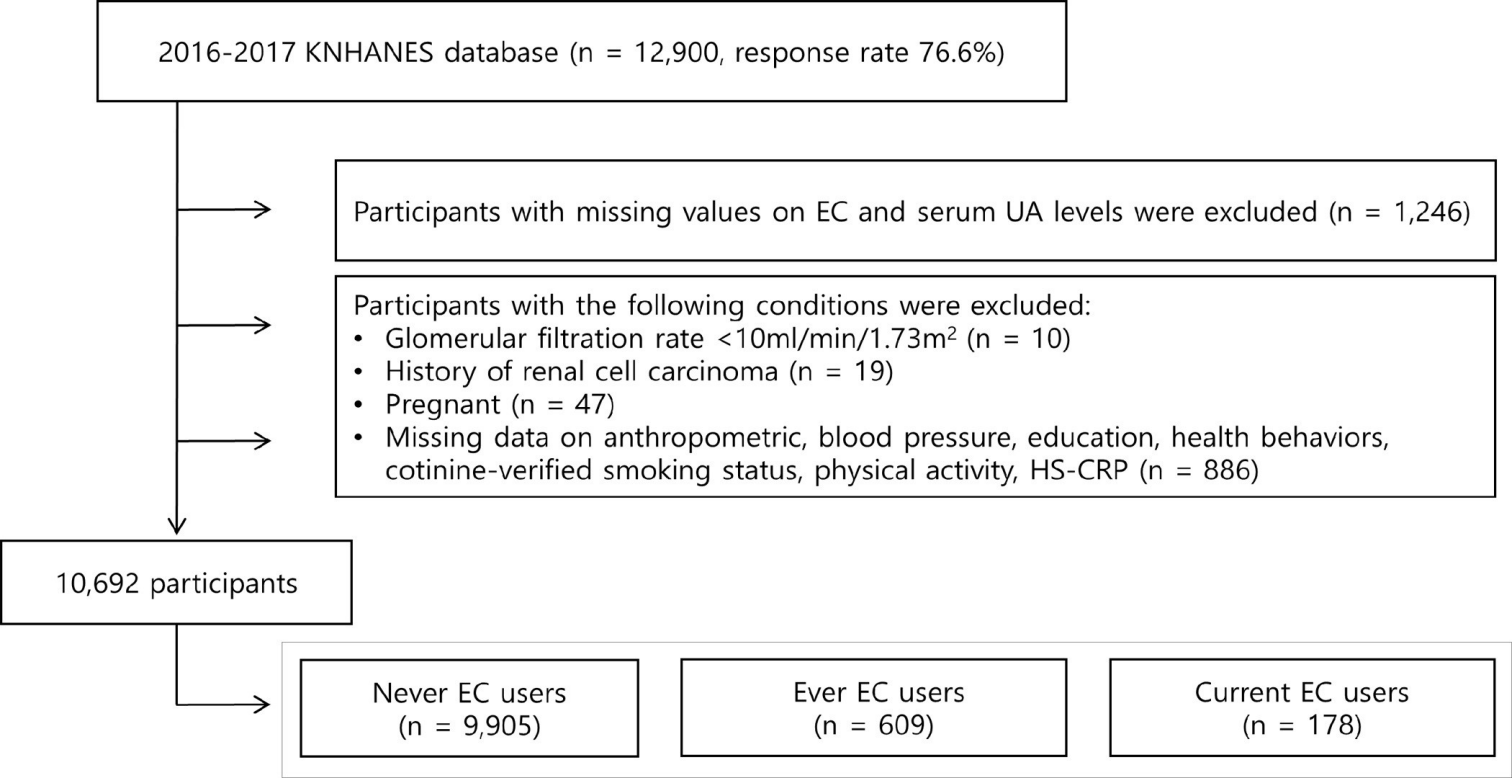
Study flow diagram.

A total of 12,900 individuals aged ≥19 years participated in the 2016–2017 KNHANES, with a response rate of 76.6%. Among them, we identified 11,654 participants with available data on EC and serum UA levels. Participants with glomerular filtration rate (GFR) <10 (n = 10), with a history of renal cell carcinoma (n = 19), or who were pregnant (n = 47) were excluded from the analysis. Subsequently, 886 participants with missing data on anthropometric (n = 11), blood pressure (n = 13), education (n = 412), health behaviors (alcohol consumption [n = 6], cotinine-verified smoking status [n = 281], or physical activity [n = 33]), and high sensitive C-reactive protein (HS-CRP, n = 130) level were also excluded from the study. Finally, 10,692 participants (9,905 never, 609 ever, and 178 current EC users, respectively) were included in the analysis. The study flow chart is summarized in Fig 1.

The study protocol was approved by the Kosin University Gospel Hospital Institutional Review Board (IRB, No. 2019-11-033). All study procedures complied with the tenets of the Declaration of Helsinki and STrengthening the Reporting of OBservational studies in Epidemiology (STROBE) guidelines. Written informed consent was obtained from all individuals.

### Data collection

EC use was classified into three groups according to responses to the following questions: (1) “Have you ever used an electronic cigarette in your lifetime?” and (2) “Have you used electronic cigarettes within the last month?” Participants responding “no” to both questions were classified as never EC users. Those who answered “yes” to the first question and “no” to the second were classified as ever EC users. Finally, participants who answered “yes” to both questions were classified as current EC users. Urine-cotinine, a metabolite of nicotine, and NNAL, which reflects objective exposure to tobacco products, were used to determine conventional smoking exposure among these groups. Among cotinine-verified active smokers, participants who consumed ECs concurrently were defined as dual users.

Smoking status was categorized as none, passive, and active based on World Health Organization (WHO) classification and urinary cotinine levels. Active smokers were defined as participants who had smoked ≥100 cigarettes in their lifetimes and who currently smoked. Passive smokers were defined as those who did not smoke but who had been exposed to tobacco smoke either in the workplace or at home during the past week. Non-smokers were those participants who had smoked <100 cigarettes in their lifetimes, were not currently smoking, and had not been exposed to second-hand environmental smoke. Because of the unreliability of self-reported smoking status, we verified each subject’s smoking status using urinary cotinine concentrations. For example, participants classified as non-smokers based on questionnaire responses with urinary cotinine levels >5 or >50 ng/mL were re-classified as passive or active smokers, respectively [25]. However, the smoking status of active and passive smokers was determined based on self-reports regardless of cotinine levels.

Information regarding socioeconomic characteristics (residential area and education) and health behaviors (alcohol consumption and physical activity) was collected in face-to-face interviews. Residential area was categorized into two groups (rural and urban). Educational level was categorized into three groups (middle school or less, high school, and college or higher). High alcohol consumption was defined as ≥7 drinks in men and ≥5 drinks in women per occasion and further categorized into two groups (less than one or more than one time per week). Physical activity was defined as moderate-intensity activity ≥150 minutes per day or ≥75 minutes of vigorous activity per week based on the WHO recommendations and then categorized into two groups (yes or no).

Blood samples were collected from the participants by skilled medical assistants and transported to the Central Laboratory (NEODIN Medical Institute, Seoul, South Korea). Serum UA (mg/dL) was measured using a Hitachi Automatic Analyzer 7600–210 (Hitachi Medical Corporation, Tokyo, Japan) by colorimetry (uricase). HUA was defined as serum urate levels >7.0 mg/dL in men and >6.0 mg/dL in women [26]. Serum creatinine (mg/dL) was also measured using a Hitachi Automatic Analyzer 7600–210 (Hitachi Medical Corporation, Tokyo, Japan). GFR was estimated using the Chronic Kidney Disease Epidemiology Collaboration (CKD-EPI) equation [27]. HS-CRP (mg/L) was measured by immune-turbidimetry using a Cobas (Roche, Bayern, Germany). Urinary NNAL (pg/mL) was measured by high-performance liquid chromatography (HPLC) with tandem mass spectrometry (MS/MS) using an Agilent 1200 Series with Triple Quadrupole 5500 (AB Sciex, Foster City, CA, USA). Urine cotinine concentration (ng/mL) was measured by HPLC-MS/MS using an Agilent 1100 Series with API 4000 (AB Sciex, Foster City, CA, USA).

Blood pressure (mmHg) was measured using a standard mercury sphygmomanometer (Baumanometer Wall Unit 33(0850); Baum Co., Inc., Copiague, NY, USA). All individuals were requested to rest in a comfortable position for at least 5 minutes. Trained medical assistants performed measurements three times and the mean of the latter two values was used in the analysis. Quality control and assurance of blood pressure measurement were periodically performed by the Korean Association of External Quality Assessment Service.

Anthropometric variables were measured by trained medical assistants at the mobile examination center. Body mass index (BMI) was calculated as kg/m^2^ and categorized into three groups (<23.0, 23–24.9, or ≥25 kg/m^2^).

### Statistical analysis

The KNAHNES followed a multi-stage clustered probability design to obtain a nationally representative sample of the Korean population. To address the complex survey design, non-responders, and post-stratification census data-based sample weights were determined. Therefore, all analyses applied complex survey design and sample weights.

The general characteristics of the study participants were compared according to EC use (never, ever, or current users). One-way analysis of variance was used for normally distributed continuous variables. Other continuous variables which violated normality (HS-CRP, urinary cotinine, and NNAL) were compared using Kruskal–Wallis tests. Subsequently, Mann–Whitney U tests were performed for multiple comparisons. Because the KNHANES measured NNAL levels in half of the samples by random selection, NNAL concentrations from 4,050 participants were analyzed.

The association between EC use and serum UA levels was evaluated using a general linear regression model. Variables with *P* <0.1 in the univariate analysis or significantly associated with HUA in earlier studies were selected as covariates for adjustment. To improve normality, log-transformed HS-CRP was included in the analyses. Thus, Model 1 was adjusted for age; Model 2 was additionally adjusted for BMI and GFR; and Model 3 additionally adjusted for residence, education, smoking status, alcohol consumption, physical activity, blood pressure, and HS-CRP. We calculated the *P-*values for the trends by using EC as a continuous variable in the analysis model. A multivariate logistic model was used to estimate the associations between EC and HUA after stepwise adjustment for the above-mentioned covariates.

In addition, as most current EC users were also active smokers, we conducted subgroup analysis among cotinine-verified active smokers to compare the difference between dual use and CC only use. The association between EC, serum UA level and the OR of HUA was measured using a general linear regression model and multivariate logistic model, respectively. Furthermore, because the primary focus of the present study was to measure the association between EC and HUA, an unweighted analysis was performed to determine whether analyses without sample weighting and clustering altered the association observed in the weighted analysis.

All tests were two-tailed and *P* <0.05 was considered statistically significant. All statistical analyses were performed using IBM SPSS Statistics for Windows, version 24.0 (IBM Corp., Armonk, NY, USA).

## Results

The general characteristics of study participants are summarized in Table 1. The present study included 10,692 participants (9,905, 609, and 178 never, ever, and 178 current EC users, respectively). Statistically significant differences between the three groups were observed in age, sex, education, smoking status, alcohol consumption, BMI, systolic and diastolic blood pressure, and GFR. Never EC users were older than ever and current EC users. Men used more EC than women. Ever and current EC users were highly educated compared to never EC users. Smoking status differed significantly between the three groups, with the highest prevalence of non-CC use among never EC users and the highest prevalence of active CC use in current EC users. Ever and current EC users consumed more alcohol than never EC users.

**Table 1:** General characteristics of the study participants according to electronic cigarette use.

	Total (N = 10,692)	Never use (n = 9,905)	Ever use (n = 609)	Current use (n = 178)	*P*-value
Age (years), mean (SE)	46.9 (0.3)	48.1(0.3)	36.6(0.5)	36.2(1.0)	<0.001*^†^
Sex, %(SE)					<0.001*^†^
Male	51.0 (0.5)	46.9 (0.5)	90.3 (1.3)	87.0 (2.6)	
Female	49.0 (0.5)	53.1 (0.5)	9.7 (1.3)	13.0 (2.6)	
Residential area, % (SE)					0.631
Urban	84.8 (1.7)	84.6 (1.7)	86.2 (2.5)	86.0 (3.2)	
Rural	15.2 (1.7)	15.4 (1.7)	13.8 (2.5)	14.0 (3.2)	
Education, % (SE)					<0.001*^†^
Middle school	14.6 (0.6)	15.8 (0.6)	3.7 (0.7)	3.1 (1.1)	
High school	9.1 (0.4)	9.5 (0.4)	5.3 (0.9)	3.0 (1.3)	
College or higher	34.6 (0.7)	33.3 (0.7)	45.7 (2.3)	49.5 (4.3)	
Unanswered	41.7 (1.0)	41.3 (1.0)	45.3 (2.3)	44.4 (4.3)	
Smoking status					<0.001*^†^^‡^
None	62.0 (0.7)	67.9 (0.7)	9.0 (1.2)	1.1 (0.8)	
Passive	12.2 (0.4)	13.2 (0.5)	3.9 (0.8)	1.6 (1.0)	
Active	25.8 (0.6)	18.9 (0.6)	87.1 (1.5)	97.4 (1.2)	
Alcohol consumption, % (SE) ^a^					<0.001*^†^
<1/week	77.7 (0.5)	80.2 (0.5)	54.0 (2.5)	56.5 (4.1)	
≥1/week	22.3 (0.5)	19.8 (0.5)	46.0 (2.5)	43.5 (4.1)	
Physical activity, % (SE) ^b^					0.426
No	52.5 (0.7)	52.7 (0.8)	50.5 (2.4)	48.8 (4.0)	
Yes	47.5 (0.7)	47.3 (0.8)	49.5 (2.4)	51.2 (4.0)	
Body mass index (kg/m^2^), % (SE)					<0.001*^†^
<23.0	41.7 (0.6)	42.8 (0.7)	32.4 (2.0)	29.2 (3.8)	
23–24.9	22.9 (0.5)	23.0 (0.5)	22.6 (1.9)	20.5 (3.1)	
≥25	35.4 (0.6)	34.2 (0.6)	45.0 (2.3)	50.3 (4.1)	
Systolic blood pressure, mmHg	117.7 (0.2)	117.8 (0.3)	116.4 (0.6)	116.0 (0.9)	0.007*
Diastolic blood pressure, mmHg	76.0 (0.2)	75.8 (0.2)	77.8 (0.4)	77.3 (0.7)	<0.001*
Glomerular filtration rate (GFR), mL/min/1.73m^2^	97.1 (0.3)	96.4 (0.3)	102.6 (0.6)	104.5 (1.3)	<0.001*^†^
High-sensitivity C-reactive protein (HS-CRP), ng/mL	1.18 (0.0)	1.16 (0.0)	1.28 (0.1)	1.40 (0.2)	0.219
Median HS-CRP, ng/mL	0.56	0.54	0.60	0.64	
Inter-quantile range	0.36–1.07	0.35–1.06	0.40–1.20	0.40–1.15	

Data were presented as weighted percentages (standard error [SE]) or weighted means (SE) unless otherwise stated.

*P*-values calculated by Chi-square tests for categorical variables, Student’s t-tests for continuous variables, and Mann–Whitney U-tests for non-normally distributed variables.

Three post-hoc analyses with Bonferroni correction were performed, with *P* <0.017 considered significantly;

*Never vs. Ever,

†Never vs. Current,

‡Ever vs. Current.

Smoking status was categorized from self-report and urinary cotinine level (>5 ng/dL for passive smokers and >50ng/dL for active smokers).

^a^ Alcohol consumption was defined as ≥7 drinks in men and ≥5 drinks in women per occasion.

^b^ Physical activity was defined as ≥150 min of moderate activity per day or ≥75 min of vigorous activity per day based on World Health Organization (WHO) recommendations.

**Fig 2:**
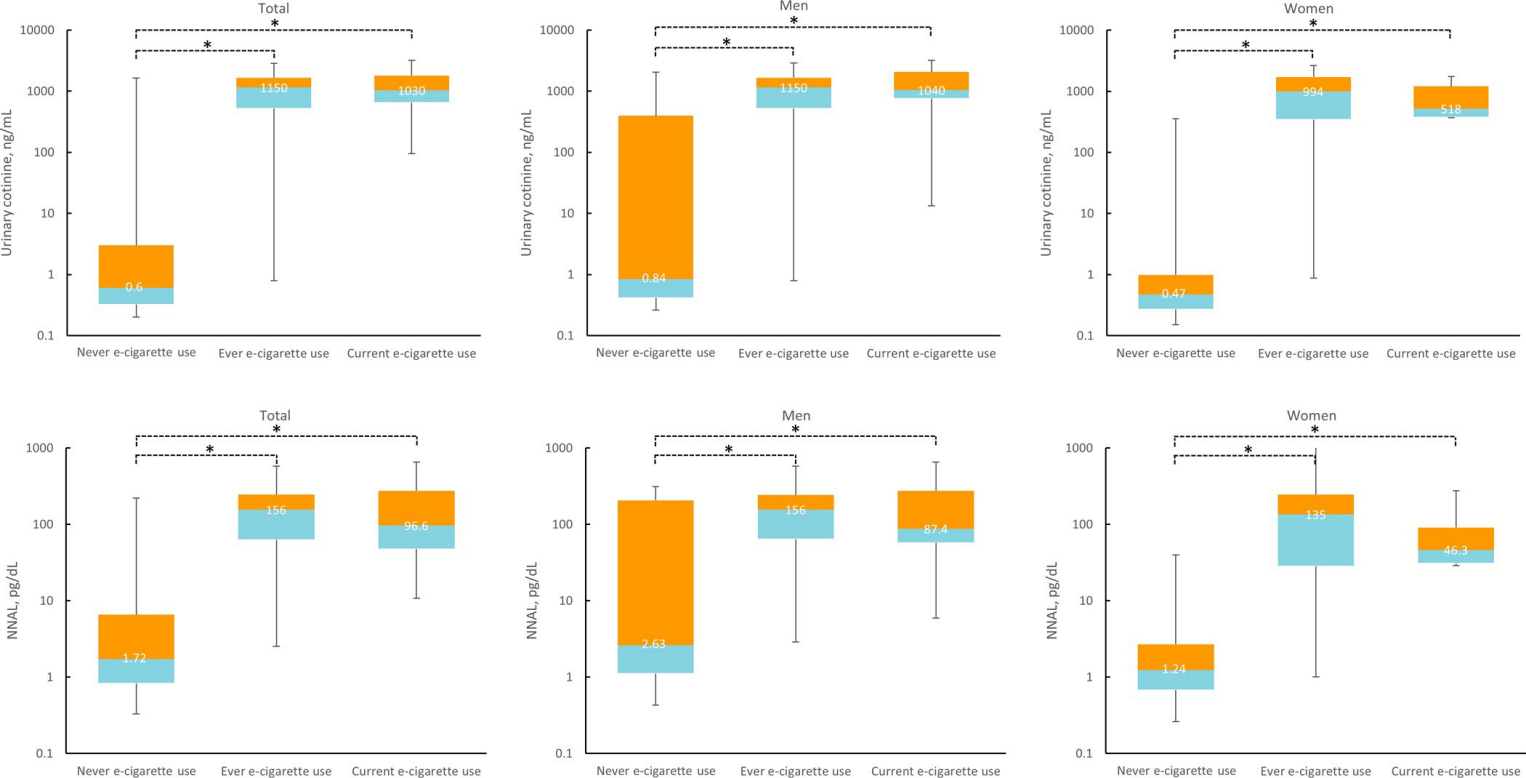
Comparison of urinary cotinine and 4-(methylnitrosamino)-1-(3-pyridyl)-1-butanol (NNAL) levels according to electronic cigarette use. Data were presented with median and interquartile range of urinary cotinine and NNAL. *P <0.05.

Comparisons of urinary cotinine and NNAL levels are shown in Fig 2. Substantial differences in urinary cotinine and NNAL levels were observed in never vs. ever EC users and never vs. current EC users, regardless of sex (*P* <0.001). However, although female current EC users had slightly lower urinary cotinine and NNAL levels compared to female ever EC users, no significant difference was observed between ever and current EC users.

**Fig 3:**
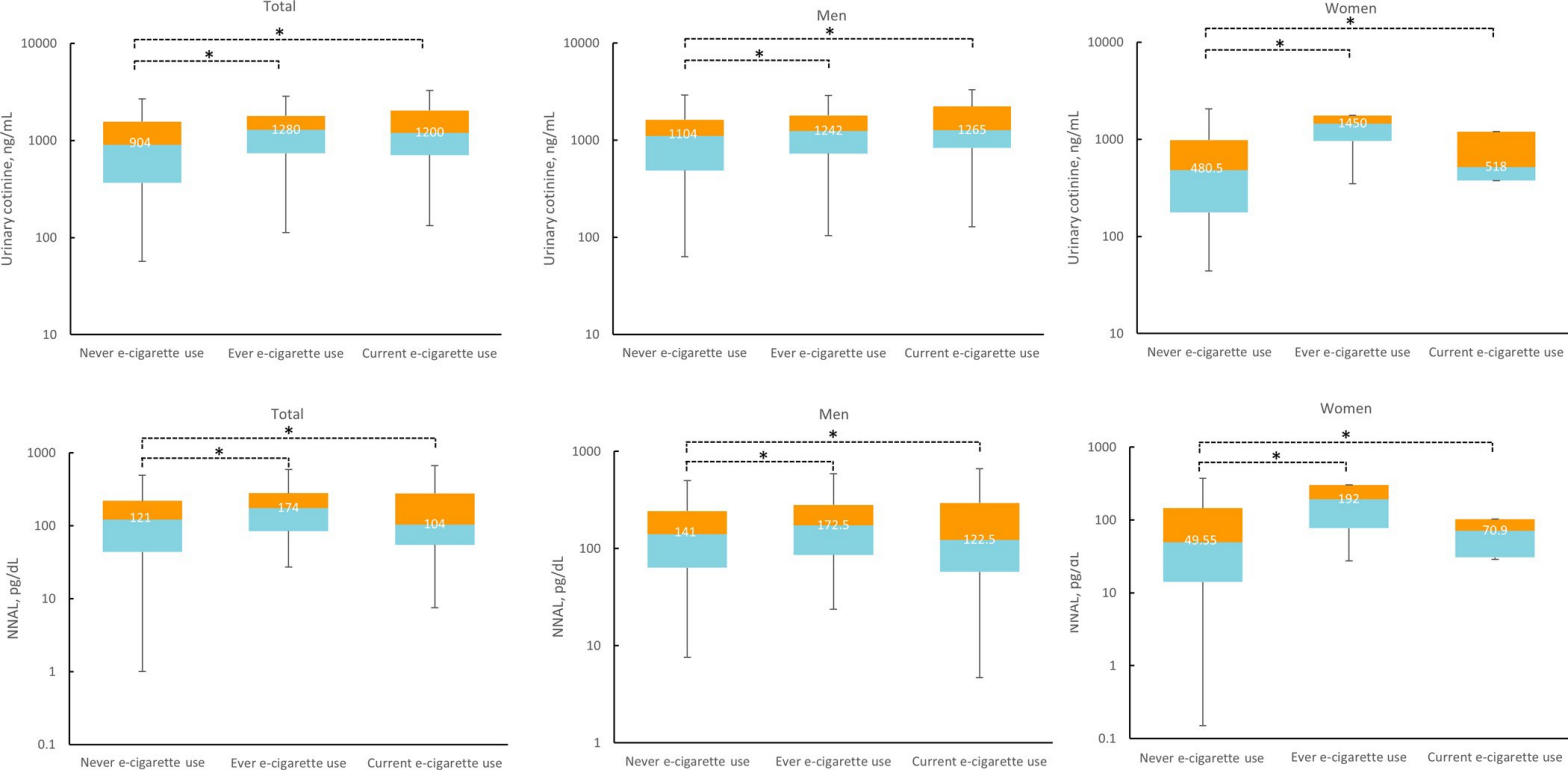
Comparison of urinary cotinine and 4-(methylnitrosamino)-1-(3-pyridyl)-1-butanol (NNAL) levels according to electronic cigarette use among urinary-cotinine verified active smokers (n = 2,361).

Fig 3 shows urinary cotinine and NNAL levels of ever and current EC users with never EC users within the subgroup of urinary-cotinine verified active CC users. Similarly in general population, significant differences in urinary cotinine and NNAL levels were observed in never vs. ever EC users and never vs. current EC users, regardless of sex.

The associations between EC use and serum UA level are presented in Table 2 with mean UA levels and 95% confidence intervals. Serum UA levels were elevated with increased EC exposure; this dose-dependent relationship remained after adjusting for potential confounders (*P*_trend_ = 0.013 in men and *P*_trend_ = 0.007 in women). Scatter plots showing the correlation between serum UA level and urinary cotinine and NNAL levels are shown in S1 Fig.

**Table 2:** Associations between electronic cigarette use and serum uric acid level among the Korean general population (N = 10,692).

	Never use	Ever use	Current use	*P*_trend_
Total				
Model 1^a^	5.12 (5.09,5.16)	5.32 (5.20,5.43)	5.44 (5.25,5.63)	<0.001
Model 2^b^	5.15 (5.12,5.18)	5.30 (5.18,5.41)	5.43 (5.24,5.62)	<0.001
Model 3^c^	5.18 (5.12,5.23)	5.33 (5.20,5.45)	5.46 (5.26,5.67)	0.001
Men				
Model 1^a^	5.88 (5.83,5.93)	5.97 (5.82,6.09)	6.07 (5.86,6.27)	0.040
Model 2^b^	5.86 (5.81,5.91)	5.94 (5.82,6.06)	6.04 (5.84,6.24)	0.044
Model 3^c^	5.86 (5.86,5.94)	5.97 (5.83,6.11)	6.09 (5.86,6.31)	0.013
Women				
Model 1^a^	4.38 (4.35,4.41)	4.74 (4.49,4.99)	5.04 (4.55,5.53)	<0.001
Model 2^b^	4.44 (4.40,4.47)	4.76 (4.51.5.02)	5.11 (4.60,5.61)	0.001
Model 3^c^	4.53 (4.47,4.59)	4.77 (4.51,5.02)	5.06 (4.57,5.56)	0.007

Data were presented as estimated mean level with 95% confidence interval.

*P*_trend_ was calculated using linear regression analysis with electronic cigarette smoking status as a continuous variable

^a^ Model 1 was adjusted for age;

^b^ Model 2 was additionally adjusted for body mass index and glomerular filtration rate;

^c^ Model 3 was additionally adjusted for residence, education, smoking status, alcohol consumption, physical activity, blood pressure, and high-sensitivity C-reactive protein.

Table 3 shows similar association between EC use and serum UA levels within urinary-cotinine verified active smokers. Serum UA levels were elevated with increased EC exposure; this dose-dependent relationship remained after adjusting for potential confounders (*P*_trend_ = 0.004 in men and *P*_trend_ = 0.020 in women).

**Table 3:** Association between electronic cigarette use and serum uric acid level among urinary cotinine-verified active smokers (n = 2,361).

	Never use	Ever use	Current use	*P*_trend_
Total				
Model 1^a^	5.16(5.09,5.23)	5.36(5.22,5.50)	5.52(5.33,5.72)	<0.001
Model 2^b^	5.24(5.16,5.31)	5.38(5.24,5.52)	5.56(5.36,5.76)	0.001
Model 3^c^	5.29(5.18,5.39)	5.44(5.29,5.59)	5.62(5.41,5.83)	0.001
Men				
Model 1^a^	5.82(5.73,5.90)	5.99(5.85,6.14)	6.15(5.95,6.35)	0.001
Model 2^b^	5.83(5.74,5.91)	5.96(5.82,6.09)	6.12(5.92,6.32)	0.008
Model 3^c^	5.83(5.0,5.96)	5.97(5.81,6.13)	6.14(5.92,6.37)	0.004
Women				
Model 1^a^	4.50(4.38,4.61)	4.74(4.42,5.05)	5.00(4.49,5.51)	0.030
Model 2^b^	4.57(4.45,4.69)	4.81(4.50,5.12)	5.12(4.58,5.65)	0.024
Model 3^c^	4.69(4.52,4.87)	4.95(4.63,5.27)	5.17(4.66,5.68)	0.020

Data were presented as estimated mean level with 95% confidence interval.

*P*_trend_ was calculated using linear regression analysis with electronic cigarette smoking status as a continuous variable

^a^ Model 1 was adjusted for age;

^b^ Model 2 was additionally adjusted for body mass index and glomerular filtration rate;

^c^ Model 3 was additionally adjusted for residence, education, smoking status, alcohol consumption, physical activity, blood pressure, and high-sensitivity C-reactive protein.

Table 4 shows the associations between EC use and HUA among the Korean general population. The prevalence of HUA was 26.2%, 19.3%, and 10.8% in current, ever, and never EC users, respectively. EC use was significantly associated with HUA in a dose-dependent manner in men (*P*_trend_ = 0.041). However, although a similar trend was also observed in women, this association did not reach statistical significance (*P*_trend_ = 0.102). Compared to never EC users, the OR of HUA was 1.72 (95% CI: 1.13–2.60) in male current EC users and 1.10 (95% CI: 0.81–1.50) in male ever EC users (*P*_trend_ = 0.041). In women, the OR of HUA was 4.12 (95% CI: 0.92–18.44) in current EC users and 1.28 (95% CI: 0.42–3.89) in ever EC users, compared to never EC users (*P*_trend_ = 0.102).

**Table 4:** Associations between electronic cigarette use and hyperuricemia among the Korean general population (N = 10,692).

	Never use	Ever use	Current use	*P*_trend_
Total				
Prevalence of hyperuricemia, %	10.8 (0.4)	19.3 (2.0)	26.2 (3.5)	<0.001
Model 1^a^	Reference	1.20 (0.92,1.56)	1.84 (1.28,2.66)	0.002
Model 2^b^	Reference	1.14 (0.86,1.52)	1.86 (1.26,2.76)	0.006
Model 3^c^	Reference	1.10 (0.81,1.50)	1.82 (1.19,2.77)	0.023
Men				
Prevalence of hyperuricemia, %	17.0 (0.7)	20.5 (2.1)	27.7 (3.7)	<0.001
Model 1^a^	Reference	1.06 (0.80,1.40)	1.57 (1.08,2.29)	0.059
Model 2^b^	Reference	1.04 (0.78,1.39)	1.59 (1.09,2.33)	0.073
Model 3^c^	Reference	1.08 (0.79,1.49)	1.72 (1.13,2.60)	0.041
Women				
Prevalence of hyperuricemia, %	5.4(0.4)	7.9 (3.1)	16.1 (9.6)	<0.001
Model 1^a^	Reference	2.15 (0.90,5.12)	5.07 (1.21,21.18)	0.009
Model 2^b^	Reference	2.04 (0.76,5.46)	6.55 (1.42,30.17)	0.009
Model 3^c^	Reference	1.28 (0.42,3.89)	4.12 (0.92,18.44)	0.102

Data were presented as percentages with standard error or odds ratios with 95% confidence interval.

*P*_trend_ was calculated using linear regression analysis with electronic cigarette smoking status as a continuous variable.

^a^ Model 1 was adjusted for age;

^b^ Model 2 was additionally adjusted for body mass index and glomerular filtration rate;

^c^ Model 3 was additionally adjusted for residence, education, smoking status, alcohol consumption, physical activity, blood pressure, and high-sensitivity C-reactive protein.

S1 Table shows the association between EC use and HUA in unweighted analysis. Compared to never EC users, the OR for HUA was 1.74 (95% CI: 1.18–2.59) in male current EC users and 1.20 (95% CI: 0.93–1.54) in male ever EC users (*P*_trend_ = 0.005). In women, the OR of HUA was 2.40 (95% CI: 0.65–8.83) in current EC users and 1.72 (95% CI: 0.70–4.22) in ever EC users, compared to never EC users (*P*_trend_ = 0.093).

Subgroup analysis of cotinine-verified active CC users (n = 2,361) showed a more prominent association between EC use and HUA (Table 5). The OR of HUA was 1.91 (95% CI: 1.24–2.95) in current male EC users and 1.17 (95% CI: 0.82–1.67) in ever male EC users, compared to never male EC users (*P*_trend_ = 0.011). In women, the OR of HUA was 2.94 (95% CI: 0.75–11.55) in current EC users and 0.93 (95% CI: 0.25–3.42) in ever EC users, compared to never EC users (*P*_trend_ = 0.253).

**Table 5:** Subgroup analysis of the associations between electronic cigarette use and hyperuricemia among urinary cotinine-verified active smokers (n = 2,361).

	Never use	Ever use	Current use	*P*_trend_
Total				
Prevalence of hyperuricemia, %	14.2 (0.9)	19.2 (2.1)	26.6 (3.6)	<0.001
Model 1^a^	Reference	1.24 (0.90,1.71)	1.94 (1.30,2.90)	0.002
Model 2^b^	Reference	1.15 (0.82,1.60)	1.86 (1.23,2.81)	0.011
Model 3^c^	Reference	1.17 (0.83,1.65)	1.96 (1.29,2.99)	0.006
Men				
Prevalence of hyperuricemia, %	15.8 (1.1)	20.2 (2.2)	28.1 (3.8)	<0.001
Model 1^a^	Reference	1.23 (0.88,1.73)	1.90 (1.26,2.88)	0.055
Model 2^b^	Reference	1.14 (0.81,1.62)	1.78 (1.16,2.73)	0.022
Model 3^c^	Reference	1.17 (0.82,1.67)	1.91 (1.24,2.95)	0.011
Women				
Prevalence of hyperuricemia, %	8.6 (1.6)	8.2 (3.8)	16.6 (9.9)	<0.001
Model 1^a^	Reference	1.05 (0.34,3.22)	2.40 (0.54,10.61)	0.325
Model 2^b^	Reference	1.12 (0.35,3.62)	3.48 (0.72,16.80)	0.188
Model 3^c^	Reference	0.93 (0.25,3.42)	2.94 (0.75,11.55)	0.253

Data were presented as percentages with standard error or odds ratios with 95% confidence interval.

*P*_trend_ was calculated using linear regression analysis with electronic cigarette smoking status as a continuous variable

^a^ Model 1 was adjusted for age;

^b^ Model 2 was additionally adjusted for body mass index and glomerular filtration rate;

^c^ Model 3 was additionally adjusted for residence, education, smoking status, alcohol consumption, physical activity, blood pressure, and high-sensitivity C-reactive protein.

## Discussion

To our knowledge, this is the first population-based nationwide study to address the potential association between EC use and UA level. Our findings showed that EC use was associated with elevated serum UA level in a dose-dependent manner. Notably, dual users had higher serum UA levels than CC users; this group had a higher OR for HUA compared to the never EC, only CC using group among cotinine-verified active smokers.

EC use was significantly associated with increased UA in a dose-dependent manner. The OR of HUA also increased with higher EC exposure, although the strength of association was slightly attenuated in the fully adjusted model. Similar findings were observed in unweighted analysis. This result is consistent with that of a recent cross-sectional study from middle-aged healthy Romanian adults in which the serum UA level was significantly higher in current EC users than that in never smokers (mean level: 5.24 vs. 4.36 mg/dL respectively, *P* <0.01) [21] and the UA level increased with the number of EC heets per day. Another *in vivo* study in mice [28] demonstrated a dose-dependent association between serum UA levels and nicotine concentration of EC liquid, a finding also in line with those of our study.

In all participants, urinary cotinine and NNAL levels were significantly higher in EC users (current and ever) than those in never EC users, regardless of sex. The high proportion of active CC use among ever and current EC users might be attributable to this finding. In this regard, investigation of the effects of ECs on UA levels should consider the varying prevalence of CC use among never, ever, and current EC users. However, a major challenge in identifying conventional smokers in epidemiologic studies is the inaccuracy of self-reported smoking status [23, 29]. Thus, verifying smoking status using self-reported data and biochemical methods in a complementary way is necessary to determine smoking status for accurate adjustment. Re-classified numbers of participants according to biochemical method are presented in S2 Table.

Among EC users, no significant difference in the median urinary cotinine level and NNAL concentration was observed between ever and current EC users. Because EC use was categorized based on use within the previous month, urinary cotinine and NNAL levels, which have half-lives of 16 hours and 2 weeks, respectively, might not fully reflect the degree of EC exposure [30]. Although biochemical methods to determine smoking exposure may be a more reliable way to detect hidden female smokers in Asian countries [23], these biochemical markers are limited in identifying EC use because of concurrent CC use. Interestingly, nearly 97.4% of current EC user consumed CC concurrently in our data. There is a possibility that current EC users with non-CC user misclassified as passive or active CC users, because urinary cotinine levels are similar in EC users and smokers [31].

We conducted a subgroup analysis of EC use and OR of HUA among cotinine-verified active CC users to objectively investigate the association between EC and HUA. The negative impact of EC on HUA was more apparent in cotinine-verified active smokers (OR 1.96, 95% CI 1.29–2.99) than in the general population (OR 1.82, 95% CI 1.19–2.77). This finding suggested that additional EC use among active smokers might have more deleterious effects on HUA than those among the general population. Although the statistical significance was observed only in men, a similar tendency was observed in women. The small number of EC users (N = 23) among female active smokers (N = 573) might be related to the diminished statistical power.

Interestingly, in men with active CC use, dual use of EC and CC decreased urine NNAL and increased urine cotinine levels, which suggests that dual users consume fewer CCs than never EC users but are chronically exposed to more nicotine. In a previous cohort study [32], although dual users consumed CCs less frequently than only CC users, urine cotinine levels were almost identical, suggesting that dual-users supplemented their nicotine intake via ECs. In another study, in which the frequency of CC smoking between dual users and only CC smokers was similar, the urine cotinine level was higher among dual users [33].

Although the underlying mechanism of EC in UA elevation is unclear, several possible mechanisms could explain the observed association. First, nicotine in EC components not only increase but also subsequently induce endothelial tissue inflammation. A recent randomized crossover trial reported increased myeloperoxidase level (from 13.6 to 18.9 ng/mL), a marker of inflammation, after exposure to EC with nicotine [34], suggesting that inflammation and oxidative damage could be responsible for increased UA levels. The significantly higher urinary cotinine levels among ever and current EC users compared to never EC users support this mechanism. Finally, heavy metals such as lead and cadmium in EC components might also be attributable to increased UA level. Recent epidemiologic studies showed higher serum UA levels and risk for HUA among individuals exposed to the upper limits of the reference ranges of lead and cadmium [35, 36].

Second, ultra-structural change in the kidneys induced by nicotine in EC could cause increased UA levels. An experimental study in rats demonstrated tubular damage after exposure to EC with nicotine [8]; this tubular damage might have contributed to decreased UA excretion in the kidneys [37]. In another *in vivo* study, rats exposed to nicotine via drinking water showed increased proteinuria and glomerular injury [38]. Although short-term exposure to nicotine may be associated with elevated GFR and impaired UA reabsorption, continuous signaling of nicotine receptors is likely to cause chronic kidney damage and contribute to increased serum UA levels [38, 39]. However, in the present study, current and ever EC users had higher eGFR than never EC users, which is inconsistent with the previous explanations.

The present study has a few limitations. First, because the study design did not address temporality, caution is needed when interpreting the observed associations. Second, the variations in serum UA levels were minimal. Although we adjusted for potential confounding factors including age, education, alcohol consumption, etc., unmeasured confounders that might affect the serum UA levels such as high-purine diet and use of diuretics were not considered in the analysis model. In addition, because the characteristics of study participants show striking difference in some variables, unmeasured confounders could interrupt robust causal inference. Third, detailed information on EC exposure such as the number consumed per day, pack-year, or periods of smoking cessation may have provided additional insight into the association between EC and HUA. Fourth, although many EC types and brands exist, with different chemical components, the KNHANES database did not contain information on the specific EC types. Fifth, because data on gout diagnosis were not available, we could not evaluate the effect of EC on the OR for gout. Sixth, potential misclassification of non-smoking EC users as passive or active smokers may contribute to the low numbers of current EC users (1.1%) and result in EC users being over-represented in the subgroup analysis with cotinine-verified active smokers. Finally, the effect of EC on HUA might have been overestimated, especially in women, because the magnitude of the association decreased after adjusting for smoking status in the analysis model of women.

Despite these limitations, to our knowledge, this is the first population-based study to investigate the association between EC and UA and HUA. Furthermore, biochemically verified smoking status was used to accurately adjust for CC use in assessing the effect of EC on HUA as smoking status based on self-reports could underestimate active smokers [29]. Third, our study robustly adjusted for multiple compounders to measure the independent association between EC and HUA. Fourth, higher serum UA levels and prevalence of HUA in dual users may warrant further independent cohort studies to verify the causal relationship between UA and EC use.

## Conclusion

EC exposure was associated with increased serum UA level and OR of HUA. A more prominent association between EC use and OR of HUA was observed in cotinine-verified active smokers. Our results suggested that the dual use of ECs and CCs could have a more negative impact on serum UA level. Further study is needed to elucidate the underlying mechanisms of these findings.

## Supporting information

S1 Fig Scatter plot illustrating the correlation between log-transformed urinary cotinine and NNAL levels and serum uric acid levels. (TIF)

S1 Table Unweighted analysis of electronic cigarette use and hyperuricemia among the Korean general population (N = 10,692). (DOCX)

S2 Table Comparison between self-reported smoking status and urinary-cotinine verified smoking status. (DOCX)
