# Supplementary figures and images for: Association of electronic cigarette exposure with serum uric acid level and hyperuricemia: 2016-2017 Korea National Health and Nutritional Examination Survey
Source: PLoS One. 2021 Mar 1;16(3):e0247868. doi: 10.1371/journal.pone.0247868 (PMC7920355; doi:10.1371/journal.pone.0247868)

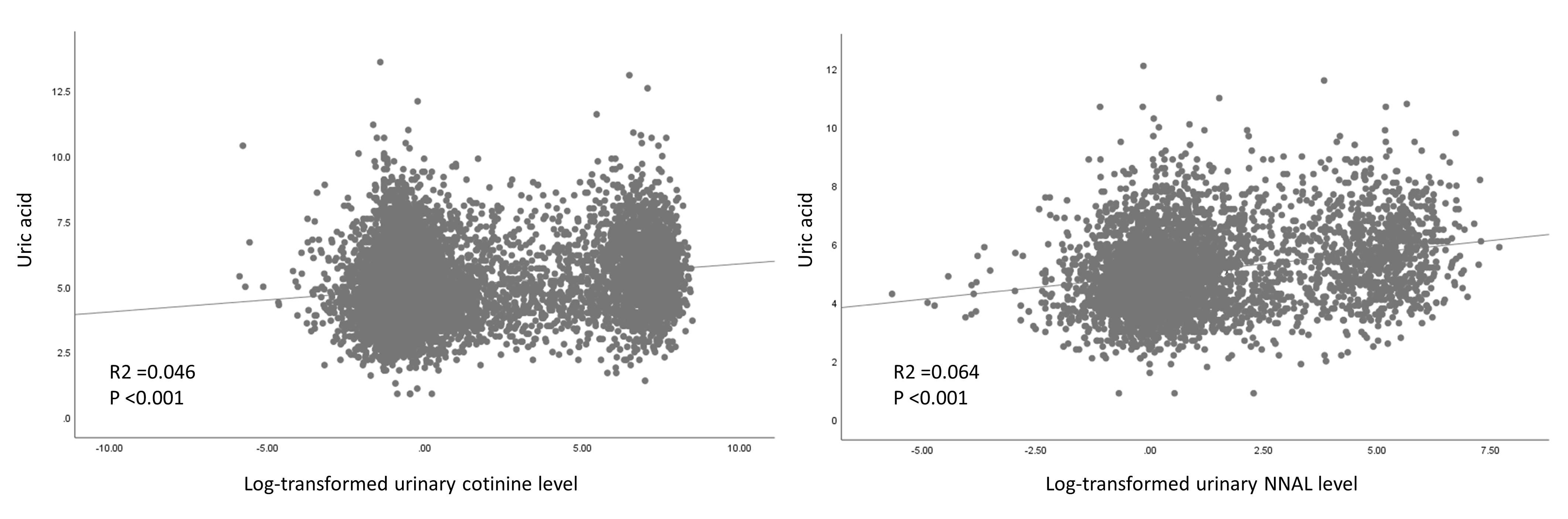

Supplement: S1 Fig — (TIF) [file pone.0247868.s001.tif]
