# Supplementary material for: Association of electronic cigarette exposure with serum uric acid level and hyperuricemia: 2016-2017 Korea National Health and Nutritional Examination Survey
Source: PLoS One. 2021 Mar 1;16(3):e0247868. doi: 10.1371/journal.pone.0247868 (PMC7920355; doi:10.1371/journal.pone.0247868)
Supplement: S1 Table — (DOCX) [file pone.0247868.s002.docx]

**S1 Table. Unweighted analysis of electronic cigarette use and hyperuricemia among the Korean general population (N = 10,692)**

|  | Never use  (n = 9,905) | Ever use  (n = 609) | Current use  (n = 178) | *P*_trend_ |
| --- | --- | --- | --- | --- |
| Total |  |  |  |  |
| Prevalence of hyperuricemia, % | 9.9% | 19.4% | 25.8% | <0.001 |
| Model 1^a^ | Reference | 1.36 (1.09,1.70) | 2.01 (1.42,2.86) | <0.001 |
| Model 2^b^ | Reference | 1.32 (1.04,1.67) | 1.95 (1.34,2.83) | <0.001 |
| Model 3^c^ | Reference | 1.20 (0.93,1.54) | 1.74 (1.18,2.59) | 0.005 |
| Men |  |  |  |  |
| Prevalence of hyperuricemia, % | 16.1% | 20.6% | 27.9% | <0.001 |
| Model 1^a^ | Reference | 1.13 (0.89,1.42) | 1.68 (1.17,2.43) | 0.008 |
| Model 2^b^ | Reference | 1.14 (0.90,1.45) | 1.67 (1.14,2.45) | 0.010 |
| Model 3^c^ | Reference | 1.15 (0.90,1.50) | 1.71 (1.14,2.60) | 0.014 |
| Women |  |  |  |  |
| Prevalence of hyperuricemia, % | 5.6% | 10.0% | 12.5% | 0.032 |
| Model 1^a^ | Reference | 2.82 (1.26,6.31) | 3.89 (1.14,13.27) | 0.001 |
| Model 2^b^ | Reference | 2.85 (1.23,6.61) | 4.74 (1.34,16.76) | 0.001 |
| Model 3^c^ | Reference | 1.72 (0.70,4.22) | 2.40 (0.65,8.83) | 0.093 |

Data were presented as percentages with standard error or odds ratios with 95% confidence interval.

*P*_trend_ was calculated using linear regression analysis with electronic cigarette smoking status as a continuous variable

^a^ Model 1 was adjusted for age; ^b^ Model 2 was additionally adjusted for body mass index and glomerular filtration rate; ^c^ Model 3 was additionally adjusted for residence, education, smoking status, alcohol consumption, physical activity, blood pressure, and high-sensitivity C-reactive protein.
