# Supplementary material for: Association of electronic cigarette exposure with serum uric acid level and hyperuricemia: 2016-2017 Korea National Health and Nutritional Examination Survey
Source: PLoS One. 2021 Mar 1;16(3):e0247868. doi: 10.1371/journal.pone.0247868 (PMC7920355; doi:10.1371/journal.pone.0247868)
Supplement: S2 Table — (DOCX) [file pone.0247868.s003.docx]

**S2 Table. Comparison between self-reported smoking status and urinary-cotinine verified smoking statue**

|  | Self-reported smoking status¶ | | |
| --- | --- | --- | --- |
|  | Non-smokers | Ex-smokers | Current smokers |
| Cotinine-verified smoking status§ |  |  |  |
| Total, n (%) |  |  |  |
| Non-smokers | 5516 (83.4) | 1531 (74.0) | 0 (0) |
| Passive smokers | 913 (13.8) | 371 (17.9) | 0 (0) |
| Active smokers | 187 (2.8) | 166 (8.0) | 2008 (100.0) |
|  |  |  |  |
| Men, n (%) |  |  |  |
| Non-smokers | 1008 (80.1) | 1367 (75.4) | 0 (0) |
| Passive smokers | 225 (17.9) | 318 (17.5) | 0 (0) |
| Active smokers | 26 (2.1) | 129 (7.1) | 1714 (100.0) |
|  |  |  |  |
| Women, n (%) |  |  |  |
| Non-smokers | 4508 (84.2) | 164 (64.6) | 0 (0) |
| Passive smokers | 688 (12.8) | 53 (20.9) | 0 (0) |
| Active smokers | 161 (3.0) | 37 (14.6) | 294 (100.0) |

¶ Based on the World Health Organization classification, non-smoker was defined as anyone who had ever smoked less than 100 cigarettes in their lifetimes or never smoked. Ex-smoker was defined as anyone who had ever smoked more than 100 cigarettes in the past and who is not a current smoker. Current smoker was defined as anyone who had ever smoked more than 100 cigarettes and who currently smoked.

§ Participants were classified as passive or active smokers when their urinary cotinine levels >5 or >50 ng/mL, respectively.
